# Indices for measurement of sustainable diets: A scoping review

**DOI:** 10.1371/journal.pone.0296026

**Published:** 2023-12-20

**Authors:** Rosa Sá de Oliveira Neta, Severina Carla Vieira Cunha Lima, Lorena Lima do Nascimento, Camila Valdejane Silva de Souza, Clélia de Oliveira Lyra, Dirce Maria Lobo Marchioni, Angelo Giuseppe Roncalli da Costa Oliveira

**Affiliations:** 1 Postgraduate Program in Collective Health, Federal University of Rio Grande do Norte, Natal, Rio Grande do Norte, Brazil; 2 Department of Nutrition, Federal University of Rio Grande do Norte, Natal, Rio Grande do Norte, Brazil; 3 Department of Nutrition, Faculty of Public Health, University of São Paulo, São Paulo, São Paulo, Brazil; University of Udine: Universita degli Studi di Udine, ITALY

## Abstract

**Introduction:**

The current food system is associated with negative impacts on health, food insecurity and environmental harm. Sustainable diets have attracted increasing interest and novel proposals with a global scope have emerged. This scoping review aims to give an overview of the analysis of all the available evidence related to the sustainable diet indices that have been developed based on the EAT-Lancet Commission.

**Methods:**

Searches were conducted in the *PubMed*, *Embase*, *Web of Science*, *Scopus* and *Science Direct* databases. This review was conducted following the PRISMA-ScR guidelines. The target population were studies addressed the use of an index or metric for assessing sustainable diets based on the EAT-Lancet Commission Summary Report were included. PCC acronym was used in the design of the study to describe eligibility criteria: P (Population)—Indexes; C (Concept)—Sustainable diets; C (Context)—Knowledge on the structure and applicability of measurement indices of sustainable diets based on EAT-Lancet recommendations available in the literature. Study eligibility criteria were restricted to papers published in English, from January 2019 through October 2022, with no population restriction.

**Results:**

A total of 1,458 papers were retrieved, 14 of which were included in the review. Seven measures of sustainable diets were identified as follow: EAT-Lancet diet score (ELD-I), New EAT-Lancet diet score (EAT), Planetary Health Diet Index (PHDI), Sustainable Diet Index (SDI), Sustainable-HEalthy-Diet (SHED), novel Nutrient-Based EAT index (NB-EAT) and World Index for Sustainability and Health (WISH). Most studies were conducted in developed countries, where greater adherence to this type of diet was found. Estimated greenhouse gas emissions was the most reported indicator of sustainability, followed by diet quality and the benefits of sustainable diets with regards to health outcomes.

**Discussion:**

We identified barriers that hinder progress towards sustainable diets, including the difficulty of comparing different indices and the tendency to neglect social aspects and the lack of common definitions and metrics. Despite being challenge, we highlight the importance of using indices that assess sustainable diets that harmonize various indicators, as recommended by the EAT-Lancet Commission, in order to promote positive changes towards a more sustainable future.

## 1. Introduction

The intrinsic relationship between nutrition and the environment is well established, as eating patterns affect the environment and vice versa [1, 2]. Technological advances, globalization and changes in agricultural systems have stimulated changes in the human diet [3] and the current global food system is increasingly associated with harm to health ranging from food insecurity to significant environmental losses [1]. Thus, there is a need for sustainable diets that are culturally acceptable, economically accessible, safe and healthy, that minimize negative impacts on biodiversity and ecosystems and optimize both natural and human resources [4].

In 2019, the EAT-Lancet Commission Summary Report delineated a combination of food groups and intake ranges that would enhance the multiple benefits for health and the planet. The Commission’s aim was to develop global scientific goals for achieving healthy and sustainable diets, called the "Anthropocene Diet", which were proposed to optimize human health without pushing the limits of the planet and which, if adopted, could significantly reduce the environmental impact of food production [5].

The current food production system has a significant impact on the environment, contributing around 20–30% of total greenhouse gas emissions (GHGE). In addition, approximately 24% of arable land suffers some degree of soil degradation, depending on the agricultural model adopted. Intensive land use for monoculture and livestock farming results in deforestation and alarming loss of biodiversity [6–8]. However, environmental problems are not only restricted to the food production phase; they also extend to processes included in the supply chain, such as transportation, processing and food preparation. Waste and residues generated along the food supply chain also contribute to the aggravation of these environmental problems [9].

A universal healthy reference diet (planetary health diet or EAT–Lancet diet) served as the basis for the development of indexes of sustainable diets that integrate multiples indicators as environmental, social, economic and health [3, 10–13]. The proposed diet consists of vegetables, fruits, whole grains, legumes, moderate or low amounts of seafood and poultry and little or no red meat, refined grains, added sugars and starchy vegetables [5].

This initiative sparked discussions on the need for changes in contemporary food systems through health sustainable diets [11] to reformulate a global food system, as continuing with the same attitudes is no longer an option [1] and, if nothing is done, the world runs the risk of not meeting the Sustainable Development Goals established by the member countries of the United Nations on the 2030 agenda [5, 14, 15].

Changes in dietary patterns and lifestyles of populations around the world in recent decades stimulate the need for the adoption of sustainable diets and tools to measure these diets, as recommended by the Commission which proposed that this transformation be achieved equitably and across different populations and food system contexts [16, 17]. The remodeling of concepts requires updating existing indices and the way these indices measure diets, as new recommendations have emerged with a planetary scope as reference [3, 11, 12, 18–20]. This scoping review aims to give an overview of the analysis of the available evidence related to the sustainable diet indices that have been developed based on the EAT-Lancet Commission.

## 2. Materials and methods

### 2.1 Study design

Given the complexity of sustainable healthy diets and the vast number of sustainable diet indexes proposed and reported in the academic literature, a scoping review design was adopted. As opposed to systematic literature reviews, which seek to answer a very specific set of questions, scoping reviews aim to defined as a type of study that seeks to explore the main concepts of the topic in question, to ascertain the size, scope and nature of the study, condensing and publishing the data, thus pointing out the existing research gaps [21].

The present review was conducted following the PRISMA extension for scoping review (PRISMA-ScR) [22] and followed the PRISMA checklist **(**S1 File). All information on the search, article selection process and data extraction were previously registered in the International Prospective Register of Systematic Reviews (PROSPERO: CRD42022311709).

The ‘PCC’ acronym was used in the design of the study combined with other eligibility criteria: P (Population)—Indexes; C (Concept)—Sustainable diets; C (Context)—Knowledge on the structure and applicability of measurement indices of sustainable diets based on EAT-Lancet recommendations available in the literature. This strategy was adopted to drive the research question of the scoping review as follows: What is the scientific evidence for indices that measure sustainable diets based on the EAT-Lancet recommendations?

The searches were restricted to papers published in English, from January 2019 (year the EAT-Lancet Report was published) to October 2022 (when searches were performed), with no population restriction. Papers that did not use the term “sustainable diets” or similar terms were excluded for not fulfilling the objectives of the present review. Systematic Reviews, case reports, book chapters, reports, comments, editorials, letters to the editor, theses, conference annals, papers not submitted to peer review, protocols, future projects, pre-prints and studies accepted for publication but not yet published were also excluded.

The method employed does not involve the participation of human subjects and therefore does not require the assessment of an ethics committee.

### 2.2 Search strategy/selection of studies

Two reviewers (RSON and LLN) conducted the searches independently in the following databases selected based on previous studies: PubMed, Embase, Scopus, Web of Science and Science Direct. Although not a MeSH term, the keyword “sustainable diet” was added using quotation marks to obtain a more directed search, as the variety and number of papers would be infinite in the absence of one of these terms. The following search strategy was employed in all databases: ("sustainable diet" OR "sustainable diets" OR "sustainable healthy diet" OR "sustainable healthy diets" OR "sustainable dietary pattern" OR “sustainable nutrition”) AND (“diet index” OR “diet score”) AND (diet OR “food consumption” OR “dietary intake” OR “food intake” OR eating).

Each reviewer screened the titles and abstracts and selected papers based on the inclusion criteria with the aid of the *Rayyan Intelligent Systematic Review*^®^ program for the removal of duplicates, the preselection of potentially eligible papers and the exclusion of those that did not meet the objective of the review. Divergences of opinion between the reviewers were resolved by a third reviewer (CVSS) who determined the eligibility of the paper in question. The full text of the preselected papers was analyzed by the reviewers independently for the extraction of data. Divergences of opinion were resolved by a discussion between the reviewers.

### 2.3 Data extraction

The preselected papers were submitted to full-text analysis for the selection of those that met the inclusion criteria. The *Microsoft Office Excel*^®^ and *Rayyan Intelligent Systematic Review*^®^ programs were used to register the decisions.

The following data were extracted from the papers selected for the present review: author and year of publication, country in which the study was developed, type of study, sample size, characteristics of participants (age, group and sex), sustainable diet index employed, structure and indicators of sustainable diets considered by the index and application on the outcome studied.

### 2.4 Methodological quality appraisal

The appraisal of the methodological quality and risk of bias in cohort and cross-sectional studies was performed using the Newcastle–Ottawa scale [23] and adapted Newcastle–Ottawa scale [24], respectively.

## 3. Results

### 3.1 Article selection process

**Fig 1:**
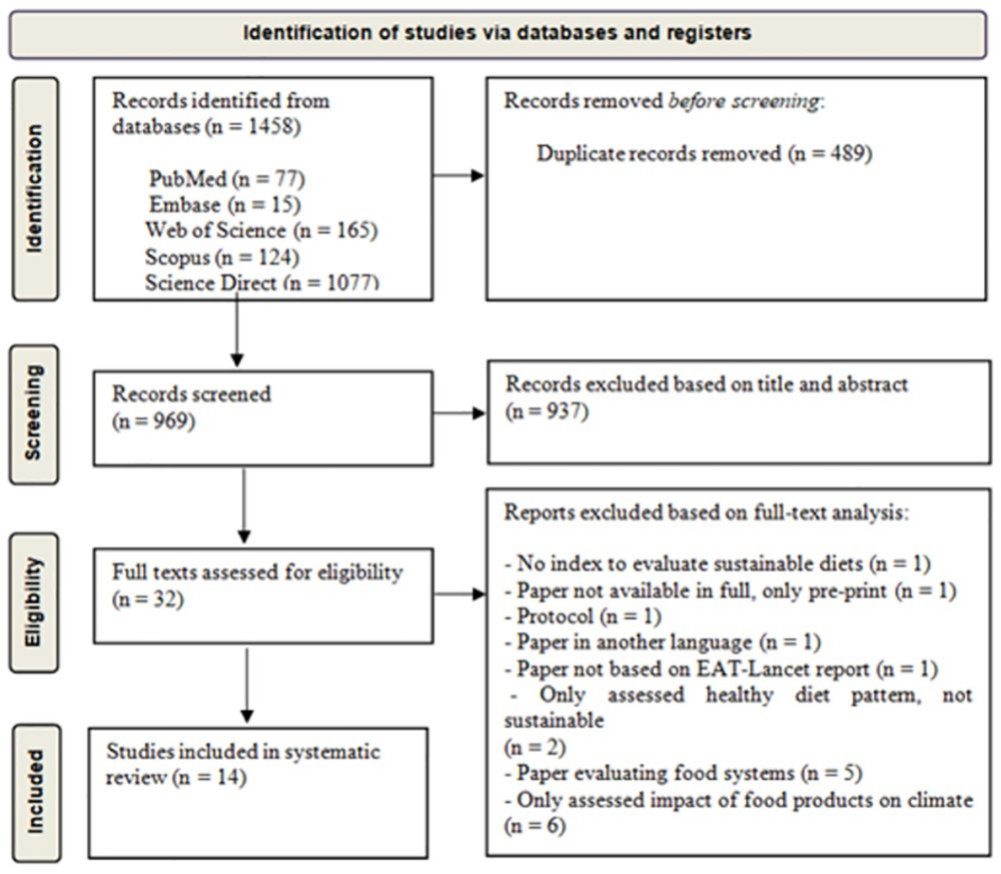
Flowchart of the study selection process.

The search of the databases led to the retrieval of 1458 papers: 77 in PubMed, 15 in Embase, 165 in Web of Science, 124 in Scopus and 1077 in Science Direct. After the removal of duplicates, the papers were screened based on the title and abstract. Incomplete papers (pre-prints), animal studies, *in vitro* studies, those focused on the transportation/distribution of food, those that offered only political analyses and surveys of public opinion were excluded. Thirty-two potentially eligible papers were submitted to full text analysis. Those that did not address sustainable diet indices and those that assessed food systems or the climatic impact of food products were excluded. Fourteen papers met the eligibility criteria and were included in the present scoping review. The evidence selection process was done as shown in the flowchart formulated based on the PRISMA recommendations [13] (Fig 1).

### 3.2 Overview and characteristics of studies

The 14 papers were conducted in 12 countries: eight in Europe (France (n = 3) [19, 25, 26], United Kingdom (n = 2) [18, 27], Sweden (n = 1) [28], Hungary (n = 1) [20] and Albania (n = 1) [29], three in the Americas Brazil [3, 30, 31], two in Asia (Israel (n = 1) [11] and Vietnam (n = 1) [12] and one multicenter study conducted in low- and middle-income countries located in South America, Africa and Asia (Democratic Republic of the Congo, Ecuador, Kenya, Sri Lanka and Vietnam) (n = 1) [32]. The aim of the studies and the sustainable diet indices employed are presented in Table 1.

**Table 1:** Overview of papers included in present scoping review (n = 14).

Author (year)	Type of study (Period/Country)	Population (size/age/origin of data)	Objective of study	Sustainable diet index	Statistical analysis/Main conclusions
Knuppel et al. (2019) [18]	Cohort (1993–2001) United Kingdom	46,069 adults European Prospective Investigation into Cancer and Nutrition	Investigate association between EAT-Lancet diet score and risk of major health outcomes.	EAT-Lancet Diet Index (ELD-I)	Cox regression/ Beneficial associations for ischemic heart disease and diabetes, but no association with stroke and no clear association with mortality.
Seconda et al. (2020) [10]	Cohort (2014–2018) France	15,626 adults Nutrinet Santé	Investigate association between sustainable eating patterns determined using SDI and risk of obesity, overweight and weight gain.	Sustainable Diet Index (SDI)	Cox regression/ Results indicate potential protective role for more sustainable diets in lower risk of weight gain, overweight and obesity.
Seconda et al. (2020) [19]	Cohort (2014–2018) France	25,592 adults (76% women) Nutrinet Santé	Investigate association between sustainable eating patterns determined using SDI and risk of cancer and cardiovascular disease.	Sustainable Diet Index (SDI)	Cox regression/ Higher SDI associated with lower risk of chronic diseases. Participants in fourth quartile (best sustainable eating patterns) had significantly lower risk of cancer and cardiovascular disease.
Llanaj et al. (2021) [29]	Cross-sectional (2015–2016) Albania	289 young adults (18–24 years) at three major universities in Albania	Examine adherence to Dietary Approaches to Stop Hypertension (DASH), EAT-Lancet reference diet (EAT), Mediterranean Diet Score (MDS) and associations with dietary cost and eating outside the home.	EAT-Lancet Diet Index (ELD-I)	Student’s t-test or Mann-Whitney U test and Poisson regression/ DASH, EAT and MDS associated with cost in neutral way. Adherence to healthy, sustainable dietary patterns was low and not differentiated by cost.
Llanaj et al. (2021) [20]	Cross-sectional (2020) Hungary	703 adults (20–64 years), 359 from general population and 344 from Romani population	Report adherence to healthy, sustainable eating patterns using score and regression models based on Dietary Approaches to Stop Hypertension (DASH) and EAT-Lancet report and determine diet quality based on Dietary Inflammatory Index (DII).	Nutrient-based EAT index (NB-EAT)	Binomial and Poisson regression/ Most NB-EAT goals not met. Results indicated high “non-conformity” with DASH pattern (95%), which could signify increased risk of diet-related chronic noncommunicable diseases and low potential for current diet to contribute to improvement in climate goals.
Kesse-Guyot et al. (2021) [25]	Cohort (2014–2018) France	29,210 adults (75% women, mean age: 53.5 years) Nutrinet Santé	Characterize environmental pressures and impacts related to degree of adherence to EAT-Lancet diet among French adults.	EAT-Lancet Diet Index (ELD-I)	ANOVA and chi-squared test/ Adherence to EAT-Lancet led to lower environmental impacts. However, some low-EAT diets (reflecting unhealthy diets) may have low environmental impacts.
Trijsburg et al. (2021) [12]	Cross-sectional (2019) Vietnam and Nigeria	396 adults (18–49 years) low-income urban population	Develop World Index for Sustainability and Health (WISH) to assess diets in terms of healthiness and sustainability.	World Index for Sustainability and Health (WISH)	Spearman’s correlation/ Higher score found for less-healthy (mean: 20 out of 30) sub-score. Initial analysis showed that WISH is able to differentiate between healthiness and environmental sustainability of Vietnamese diet.
Cacau et al. (2021) [3]	Cross-sectional (2008–2010) Brazil	14.779 adults and older people (35–74 year) *Estudo Longitudinal de Saúde do Adulto* (ELSA) (Longitudinal Adult Health Study)	Propose development of Planetary Health Diet Index (PHDI) based on EAT-Lancet reference diet.	Planetary Health Diet Index (PHDI)	Principal component analysis and multiple linear regression/ After adjustments for age and sex, PHDI score remained associated (p < 0.001) with overall diet quality and lower carbon footprint, confirming the validity and reliability of the PHDI.
Cacau et al. (2021) [31]	Cross-sectional (2008–2010) Brazil	14,515 adults and older people (35–74 years) *Estudo Longitudinal de Saúde do Adulto* (ELSA) (Longitudinal Adult Health Study)	Assess adherence to PHDI and association with obesity in ELSA study	Planetary Health Diet Index (PHDI)	Multiple linear regression/ Inverse association between adherence to PHDI and obesity indicators.
Hanley-Cook et al. (2021) [32]	Cross-sectional (2009–2015) Congo, Ecuador, Kenya, Sri Lanka and Vietnam	1,950 women in reproductive age (15–49 years)	Investigate associations between EAT-Lancet diet scores and mean probability of adequate nutrient intake among women in reproduction age and nutritional insecurity in low- and middle-income countries.	EAT-Lancet Diet Index (ELD-I)	Linear regression/ EAT-Lancet diet requires minimum intake values for nutrient-dense dietary components to avoid positively scoring non-consumption of food groups and subsequently predicting lower adequacy micronutrients in diets when applied to food insecurity.
Tepper et al. (2021) [11]	Cross-sectional Israel	348 adults (20–45 years)	Establish a practical tool that can measure and score a healthy sustainable diet	Sustainable-HEalthy-Diet (SHED)	Principal component analysis/ Significant linear correlation found between SHED index score and food groups of EAT-Lancet reference diet.
Xu et al. (2022) [27]	Cohort (2006–2010) United Kingdom	59,849 adults and older people (40–69 years) UK Biobank Cohort	Explore the association between a healthy diet pattern (EAT-Lancet, EAT-LDP) and the risk of type 2 diabetes.	EAT-Lancet Diet Index (ELD-I)	Spline cubic regression and Cos regression/ One-point increase in diet score associated with 6% reduction in risk of type 2 diabetes mellitus.
Marchioni et al. (2022) [30]	Cross-sectional (2017–2018) Brazil	46,164 adolescents, adults and older people (> 10 years of age) Family Budget Survey	Investigate adherence to EAT-Lancet diet using Planetary Health Diet Index (PHDI) in a national population-based sample.	Planetary Health Diet Index (PHDI)	Logistic regression/ Women, older people, overweight/obese individuals, those with higher income *per capita* and residents of urban areas had higher PHDI scores. In general, Brazilian population has low adherence to healthy, sustainable dietary pattern and seems to be far from meeting the goals of EAT-Lancet.
Stubbendorff et al. (2022) [28]	Cohort (5 years) Sweden	22,421 adults and older people (45–73 years) Malmö Diet and Cancer Study (MDCS)	Develop a novel dietary index to quantify adherence to the EAT-Lancet diet and assess the association with mortality. Examine the food components included in the index and individual associations with mortality.	New EAT-Lancet Diet Index (EAT)	Cox regression/ Divided into five adherence groups; greater adherence to EAT-Lancet diet associated with lower all-cause mortality. Results indicate 25% lower risk of mortality among those with greater adherence to EAT-Lancet diet.

The papers were published between 2019 and 2022, with the greatest production in 2021 (8/14), demonstrating that interest in this field of research is recent. All papers selected were in the first or second quartile of the Journal Citation Reports and all were published in English.

Six studies had a cohort design [9, 10, 18, 25, 27, 28] and eight had a cross-sectional design [3, 11, 12, 20, 21, 27, 31, 32]. Average duration of the cohort studies was 4.8 years. Most studies were conducted with individuals or secondary national data. Only one study involved a multi-country or global approach. A total of 277,911 individuals participated in the studies included.

Adherence to sustainable diets varies around the world as different measurement indices are used making it difficult to compare across different populations. Initially in 2019, an index was developed based on the EAT-Lancet recommendations in the United Kingdom (ELD-I) in which 1 point was assigned for each recommendation met by the population out of a total of 14 points. This study evaluated cardiometabolic risk as an outcome and had scores ranging from 4 to 14 points, with beneficial associations being found for ischemic heart disease and diabetes, but no association with stroke and no clear association with mortality [18].

Xu et al. (2022) found that higher adherence to ELD-I contributes to a lower risk of diabetes [27]. Another study carried out in Albania used the same index with the aim of making associations with the cost of the diet and the place of intake (inside and outside home) and concluded that low adherence was not differentiated by cost, but by place of consumption [29]. Then, a new dietary index was developed based on ELD-I (EAT index) to investigate adherence to the EAT-Lancet diet in a Swedish cohort and its association with mortality and had adherence of, an average of 17.9/42 points was obtained [28].

Studies using the SDI as an index to assess adherence to sustainable diets found an inverse association between adherence and weight gain, overweight and obesity, supporting a potential protective role of more sustainable diets in this context [19]. When applying SDI to outcomes such as cancer and cardiovascular disease risk, greater adherence was found to be associated with a significant decrease in cancer or cardiovascular disease risk [26].

Brazilian studies that used the PHDI presented an average score of 60.4/150 [31] and 45.9/150 [30], respectively. The difference between the scores obtained using the same index may be related to the different populations analyzed. In the first study, the population was composed of federal civil servants, while the second study was conducted using data from a population-based study.

Adherence to sustainable diets when assessed in the general Hungarian population was 2.0/12 points when using a nutrient-based index (NB-EAT) [20]. This score was 5.9/20 possible points in Israel when using the SHED index [11]. When using the WISH index in Vietnamese living in urban areas, adherence was 46/130 points [12].

### 3.3 Sustainable diet measures

Seven sustainable diet measures were cited: EAT-Lancet diet score (ELD-I) (n = 5) [18, 25, 27, 29, 32], New EAT-Lancet diet score (EAT) (n = 1) [28], Planetary Health Diet Index (PHDI) (n = 3) [3, 30, 31], Sustainable Diet Index (SDI) (n = 2) [10, 18], Sustainable-HEalthy-Diet (SHED) (n = 1) [11], novel Nutrient-Based EAT index (NB-EAT) (n = 1) [20] and World Index for Sustainability and Health (WISH) (n = 1) [12]. The characteristics of these indices are described in Table 2.

**Table 2:** Characteristics of indices used in papers selected for present scoping review (n = 7).

INDEX	STRUCTURE OF INDEX	COMPONENTS OF INDEX		DIETARY ASSESSMENT METHOD	INDICATORS OF SUSTAINABLE DIET
**ELD-I**	Binary assessment (0 to 1 point) of each component of diet; Narrowest possible range: (0–14 points). Index organized into food groups. Dietary intake is assessed based on quantities reported in grams.	(1)Whole grains(< = 464g/day) and grain fibre >5g (2)Tubers and starchy vegetables (< = 100g/day) (3)Vegetables (> = 200g/day) (4)Fruits (> = 100g/day) (5)Dairy foods (< = 500g/day) Protein sources– (6) Beef, lamb, pork (< = 28g/day) (7) Chicken, other poultry (< = 58g/day) (8) Eggs(< = 25g/day) (9) Fish (< = 100g/day) Legumes– (10) Dry beans lentils, peas (< = 100g/day) (11) Soy foods(< = 50g/day) (12)Peanuts/tree nuts (> = 25g/day) (13)Added fats (unsaturated:saturated fat < = 0.8) (14) Added sugars (< = 31g/day)		Duplicate, non-consecutive 24-hour recalls were carried out for everyone and FFQ^d^.	Environmental: GHGE [18, 25, 27, 29, 32]. Quality of diet and higher consumption of vegetables and lower consumption of meat [18, 25, 27, 29, 32]. Health outcomes: Ischemic heart disease, stroke, all-cause mortality [18] and diabetes [18, 27]. Nutritional insecurity and costs [29, 32].
**EAT**	The EAT was developed based on intake levels and reference intervals of 14 food components defined in the EAT-Lancet diet (0–3 points per component; 0–42 points in total). Participant scores = 3 points if intake above target intake; 2 points = lower limit of reference range up to target intake; 1 point = 50%-100% of lower limit of reference range; 0 points = <50% of lower limit of reference range. Index organized into emphasized and limited food components. Dietary intake is assessed based on quantities reported in grams.	• *Emphasized components*: (1)Whole grains (0–60% of energy) (2)Vegetables (200-600g/day) (3)Fruits (100-300g/day) (4) Fish (0-100g/day) (5) Peanuts/tree nuts (0-75g/day) (6) Legumes (0-150g/day) (7) Unsaturated oils (20-80g/day) • *Limited components*: (8)Tubers and starchy vegetables (0-100g/day) (9)Dairy foods (0-500g/day) (10) Lamb (0-14g/day) (11) Pork (0-14g/day) (12) Chicken, other poultry (0-58g/day) (13) Eggs(0-25g/day) (14) Added sugars (0-31g/day)		1) a 7-day food diary (consecutive days) covering meals that vary from one day to the next (mainly lunch and dinner), cold drinks (including alcoholic beverages) and food supplements; 2) a 168-item FFQ^d^ covering consumption frequencies and portions of foods consumed regularly, such as breakfast and snacks, over the last 12 months and not covered in the food diary; 3) a 60-minute interview conducted to ask about cooking methods and usual portion sizes.	Environmental: GHGE [28]. Quality of diet and higher consumption of vegetables and lower consumption of meat [28]. Health outcomes: all-cause mortality, cancer mortality and cardiovascular mortality [28].
**PHDI**	Proportional assessments of each component of diet. A maximum of 10 or 5 points attributed to each of 16 components established in PHDI, resulting in a total proportional score ranging from 0 to 150 points. The higher the dietary intake of the fitness components, the higher the score obtained, with a maximum of 10 points. The optimal component foods have an ideal recommendation in which these foods have the highest score (10 points) and, if exceeded, this score is decreasing and progressive, reaching 0, the same occurs with the “ratio” components, but this group scores up to 5 points. The consumption of foods in the moderation component is discouraged, so low consumption gets the maximum score (10 points) and the minimum score if it has high consumption. Dietary intake is assessed based on quantities reported in standards for scoring (caloric densities). Index organized into food groups.	Standards for scoring in caloric densities • *Adequacy components* (0–10 points): (1) Nuts and peanuts (0–≥11.6) (2) Legumes (0–≥11.3) (3)Fruits (0–≥5.0) (4) Vegetables (0–≥3.1) (5) Whole grains (0–≥32.4) • *Optimum components* (0-10-0 points): (6) Eggs (0–0.8–≥1.5) (7) Fish, sea food (0–1.6–≥5.7) (8) Tubers and potatoes (0–1.6–≥3.1) (9) Dairy excluding dairy fats (0–6.1–≥12.2) (10) Vegetable oils (0–16.5–≥30.7) • *Ratio components* (0-5-0 points): (11) ^a^DGV/total (0–29.5–100) (12)^b^ReV/ total (0–38.5–100) • *Moderation components* (10–0 points): (13) Red meat including beef, lamb and pork (≤2.4–0) (14) Chicken and substitutes (eggs, fish or plant protein sources) (≤5.0–0) (15) Animal fat including lard, tallow and dairy fats (≤1.4–0) (16) Added sugars (≤4.8–0)		A 114-item FFQ^d^.	Environmental: GHGE [3, 30, 31]. Quality of diet and higher consumption of vegetables and lower consumption of meat [3, 30, 31]. Health outcomes: Obesity indicators [30, 31].
**SDI**	Categorized into four standardized sub-indexes, respectively, environmental, nutritional, economic and sociocultural. The index was obtained by summing the sub-indexes, which ranges from 1 to 5 points, resulting in an overall SDI score ranging from 4 to 20 points. Environmental sub-index: includes environmental indicators (Land occupation, GHGE and primary energy consumption that computed pReCiPe score), taking into account shifts from raw agricultural products until consumer use as well as the method of agricultural production (organic v. conventional). Environmental impacts of individual diets were estimated by multiplying the pReCiPe by the quantity of consumed food (g/d). Nutritional sub-index: includes two sub-scores: an adequacy sub-score assessing the probability that nutrient intake satisfied the requirements (above a reference value) and a moderate sub-score assessing the probability that nutrient intake was not excessive (over a reference value). Economic sub-index: The individual daily monetary cost of diets was computed by multiplying the food quantity consumed (g/d) by the price (€/g) after divides the total diet monetary cost by the income reported by the participants. This index consider that the affordability of diets could be assessed by the percentage of available household income for food. Sociocultural sub-index: To compute the index, two points were assigned for short supply chains defined as direct food commercialisation between producers and consumers or with only one intermediary (producers’ markets, farmers’ shops, artisans, farms and self-production) and one point for other places: markets, groceries, specialized organic shops or cooperatives. No point was attributed to supermarkets. Then the index (out of two) was obtained by summing the points and dividing by the total answers. The participants also were asked to report their frequency of consumption of canned goods, ready-made meals, and frozen foods through a 5-point ordinal scale ranging from ‘never’ to ‘always.’ Scores of 0, 0·25, 0·5, 0·75 and 1 were allocated to the corresponding modalities: never, rarely, half of time, often and always. This enabled us to calculate, for each participant, the amounts of ready-made products consumed .	**1) Nutritional—sub-index (1/5) = the sum of points × weight**		An organic semi-quantitative 264-item Food Frequency Questionnaire (Org-FFQ).	Environmental: GHGE [19, 26]. Quality of diet and higher consumption of vegetables and lower consumption of meat [19, 26]. Health outcomes: Obesity indicators [19] and risk of cancer and cardiovascular diseases [26]. Healthy and sustainable practices, support to local producers, socioeconomic aspects of food, organic food consumption, biodiversity and sociocultural aspects [19, 26].
		(1)Absolute value of difference between energy need and intake (kJ/d)–Weight 1/2 1 point: ind >4259 2 points: 4259≤ind<2849 3 points: 2849≤ind<1812 4 points: 1812≤ind <883 5 points: ind ≤883	(2) PANDiet index (1/100)–Weight 1/2 1 point: ind ≤60·7 2 points: 60·7<ind≤ 64·4 3 points: 64·4<ind≤ 68·2 4 points: 68·2<ind≤ 72·8 5 points: ind >72·8		
		**2) Environmental—sub-index (1/5) = the sum of points × weight**			
		(1)Land occupation (m2/year)–Weight 3/4 (2)GHGE (kg CO2/year) (3) Primary energy consumption (MJ/year) The three indicators are computed together in the ^c^pReCiPe 1 point: > 0·38 2 points: 0·38 ≤ind< 0·29 3 points: 0·29 ≤ind< 0·23 4 points:0·23 ≤ind <0·17 5 points: ind ≤0·17			
		(4) Contribution of organic food to diet (% weight)–Weight ¼ 1 point: ind ≤3·02 1/4 2 points: 3·02 <ind≤ 15·5 3 points: 15·5<ind≤ 30·3 4 points: 30·3<ind≤ 54·1 5 points: ind>54·1			
		**3) Economic—sub-index (1/5) = the sum of points × weight**			
		(1) Proportion of the income devoted to diet–Weight 1 1 point: ind <11·4 2 points: 16·4≤ind< 11·4 3 points: 11·4≤ind< 8·45 4 points: 8·45 ≤ind< 5·40 5 points: 5·40 ≤ind< 1·27			
		**4) Sociocultural—sub-index (1/5) = the sum of points × weight**			
		(1) Place of food purchase (1/2)–Weight ½	(2) Ready-made products (1/3)-Weight ½		
		1 point: ind <0·28 2 points: 0·28 ≤ind< 0·45 3 points: 0·45 ≤ind< 0·60 4 points: 0·60 ≤ind< 0·79 5 points: ind ≥0·79	1 point: ind ≥1·75 1/2 2 points: ind = 1·5 3 points: ind = 1·25 4 points: ind = 1·00 5 points: ind ≤1		
**NB-EAT**	Based on nutrients with binary assessment (0 to 1 point), with score ranging from 0 to 12 points according to nutrients. Adherence categories coded based on score obtained: low (0–4), moderate (5–8) and high (>8) adherence. Dietary intakes assessed based on quantities reported in grams/day.	(1) Alpha linolenic acid (≥2.5g/day) (2) Carbohydrates (≥317.3g/day) (3) Cholesterol (≤125.2mg/day) (4) Dietary fibres (≥42.9g/day) (5) Mono- and poly-unsaturated fats (≥75.9g/day) (6) Proteins (90.1g/day) (7) Saturated fats (≤22.7g/day) (8) Total fat (≤105.6g/day) (9) Calcium (≥717.8mg/day) (10) Magnesium (≥732.5mg/day) (11) Potassium (≥4100.7mg/day) (12) Added sugar (≤31.0g/day)		Duplicate, non-consecutive 24-hour recalls were carried out for everyone.	Environmental: It evaluates indirectly this indicator by considering the recommendations of the EAT-Lancet commission [20]. Quality of diet and higher consumption of vegetables and lower consumption of meat [20]. Health outcomes: adherence to antiinflamatory diet and dietary approaches to stop hypertension [20].
**WISH**	13 components scored from 0 to 10 and out of a maximum score of 130. Dietary intake assessed based on quantities reported in calories. Higher scores of individuals components and total and secondary scores indicate healthier, environmentally correct diet in the following way: **Healthy** **components includes**: Protectors components: whole grains, vegetables, fruits, dairy, fish, legumes, nuts and unsaturated oils; Neutral components: eggs, chicken and poultry; Limited components: red meat, saturated oils and added sugars. Final score to healthy diet: Summing 8 groups of protective foods and 2 neutral foods; Final score to less healthy diet: Summing 3 groups of limited foods; **Low environmental impact components includes**: whole grains, vegetables, fruits, legumes, unsaturated oils and added sugars; Final score to low environment impact: Summing 6 groups of low environmental impact foods; **High environmental impact components includes**: red meat, fish and saturated oils. Final score to high environmental impact: Summing 4 groups with moderate and three groups with high environmental impact.	(1) Whole grains ≥125g/day (100–150) (2) Vegetables 300g/day (200–600) (3) Fruits 200g/day (100–300) (4) Dairy 250g/day (0–500) (5) Red meat 14g/day (0–28) (6) Fish 28g/day (0–100) (7) Eggs 13g/day (0–25) (8) Chicken and other poultry 29g/day (0–58) (9) Legumes 75 g/day (0–100) (10) Nuts 50g/day (0–75) (11) Unsaturated oils 40g/day (20–80) (12) Saturated oils 11.8g/day (0–11.8) (13) Added sugars 31g/day (0–31)		Duplicate, non-consecutive 24-hour recalls were carried out for everyone, with a difference of at least two days between recalls.	Environmental: GHGE, land use, eutrophication, acidification, scarcity weighted water [12]. Quality of diet and higher consumption of vegetables and lower consumption of meat [12].
**SHED**	Final questionnaire based on 30-item web. Responses to items on sustainable, healthy diet recorded on Likert scale of 1 to 4 points and out of a maximum score of 120. Items classified from "almost never true" to "almost always true" or "never" to "most of the time". The SHED index is the standardized sum of the six components based on this questionnaire: Healthy eating score (10 items) Sustainable eating score (7 items) ^e^BFV score—Buy-Fruits-and-Vegetables and ready meals score consumption of frozen or refrigerated meals vs. home cooked (6 items) Water score: source of drinking water (5 items), Soda scores consumption of sugar-sweetened and low-calorie sweetened beverages (2 items)	***Healthy eating score component***: (1) Avoid ultra-processed food (2) Limit sweets and soft drinks (3) Drink mainly water (4) Prefer low sugar food (5) Avoid adding salt (6) Prefer low salt food (7) Avoid fatty meat (8) Eat food prepared days before (9) Eat 5 fruits and vegetables/day (10) Prefer plant based food ***Sustainable eating score component***: (11) Do compost (12) Consume organics products (13) Limit red meat (14) Prefer low pesticides (15) Aware of food waste (16) Buy local products (17) Follow plant-based diet ***Water score component***: (18) Drink mainly bottled sparkling water (19) Drink mainly mineral water (20) Drink mainly bottled water; (21) Drink mainly filtered water (22) Drink mainly tap water ***BFV and Ready meals score component***: (23) Consume frozen ready meals (24) Consume refrigerated ready meals (25) Prefer home cooked meals (26) Prefer self-cooked meals (27) Prefer animal based food (28) Eat out ***Soda scores consumption component***: (29) Drink artificial sweetened beverages (30) Drink sweetened beverages		A semi-quantitative 115-item FFQ^d^ items with nine frequency options, ranging from “never or less than once monthly” to “six or more times daily” self-administered electronically.	Environmental—The index does not quantify GHGE [11]. Quality of diet and higher consumption of vegetables and lower consumption of meat [11]. Healthy and sustainable practices, support to local producers, household cooking practices, food waste, bottled water consumption, ultra-processed foods consumption [11].

^a^ DGV/total ≠ ratio between energy intake of dark green vegetables (numerator) and total vegetables (denominator) multiplied by 10.

^b^ ReV/total ≡ ratio between energy intake of red and orange vegetables (numerator) and total vegetables (denominator) multiplied by 10.

^c^ pReCiPe: Recipe score = 0.0459 × GHGE (in kg CO2eq/kg) + 0.0025 × primary energy consumption (in MJ/kg) + 0.0439 × land occupation (in m2/kg).

^d^FFQ: Food Frequency Questionnaire.

^e^BFV: Buy-Fruits-and-Vegetables.

^f^GHGE: Greenhouse Gas Emissions.

### 3.4 Indicators of sustainability

**Fig 2:**
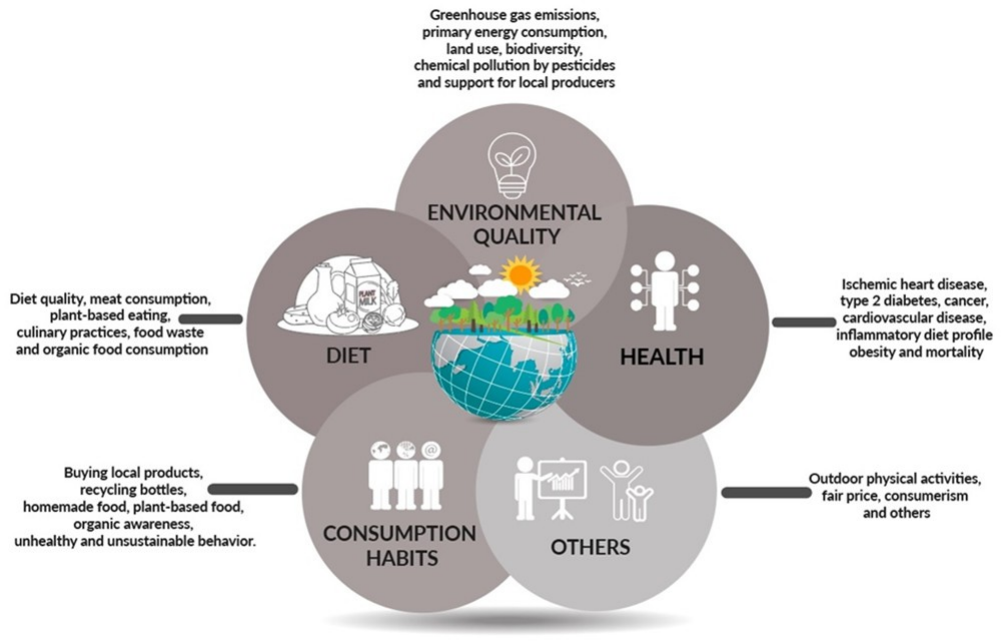
Components assessed by the sustainable diet indexes.

A total of 21 different indicators were identified. GHGE was the most measured component (n = 13; 93% of sample) [3, 10, 12, 18, 20, 25, 26–32], followed by indices that assessed diet quality with a greater consumption of vegetables and lower meat intake (n = 12; 86% of sample) [3, 11, 12, 18, 20, 25, 27–32]. Other aspects investigated were the benefits of sustainable diets with regards to health outcomes (n = 9; 62% of sample) [3, 18–20, 26–28, 30, 31], food insecurity and costs related to food (n = 4; 29% of sample) [19, 26, 29, 32] and the consumption of organic foods (n = 3; 21% of sample) [11, 19, 26]. Primary energy intake, land occupation, biodiversity, chemical pollution by pesticides, price, support to local producers, household cooking practices, food waste, bottled water consumption and ultra-processed foods were also cited, but in less than 25% of the studies (n = 3) [11, 19, 26]. No investigations were conducted of changes in transportation habits. Components assessed by the sustainable diet indexes can be seen in Fig 2.

### 3.5 Methodological quality appraisal

Risk of bias in the included studies was assessed by two independent reviewers using the Newcastle‐Ottawa Scale (NOS) for cohort studies [23] and cross-sectional studies (Modified Newcastle-Ottawa Quality Assessment Scale criteria) [24]. The Newcastle-Ottawa is a statistical tool used for assessing the quality of studies included in systematic reviews.

Each study is judged on eight items, categorized into three groups: 1) selection of the study groups (representativeness of exposed, selection of non-exposed, ascertainment of exposure, outcome not present at start); 2) comparability of the groups (control for confounders); and 3) ascertainment of either the exposure or outcome of interest (assessment of outcomes, follow-up length, adequacy of follow-up). Quality levels are either good, fair or poor. These levels are classified according to a specific score which ranges from zero to nine stars for each cohort article and ranges from zero to eight stars for each cross-sectional study, in which a greater number of stars indicates a higher‐quality study. The results can be viewed in S2 and S3 Files.

## 4. Discussion

The present scoping review investigated different tools that identify sustainable diets, which are fundamental for analyzing sustainability, establishing goals and following up the evolution of the subject as well as political decision making on the local, national and planetary levels. In our study, we found that the indices that measure sustainable diets present robust evidence in relation to what is recommended by the EAT-Lancet Commission. They are multidimensional and consider various sustainability indicators (environmental, health, economic and social); most of them allow for a gradual score that includes all the intermediate values of individual food consumption, favoring a better distinction between the degrees of adherence of individuals. In addition, they also consider interchangeable food groups, allowing adaptations to different populations, as recommended by the reference diet.

There has been an evolution over time in the development of indices to assess sustainable diets, with indicators and data used to assess the sustainability of diets adapted to each specific territory. When discussing sustainable diets, it is essential to promote an inclusive and sensitive approach to local particularities, considering the food culture, production systems, socio-economic characteristics and environmental impacts specific to each place. In this way, we can develop effective and tailored strategies to promote food sustainability in different geographical contexts [5, 6, 33]. Adherence to health and sustainable diets varied according to the population studied and assessment methods employed. Countries with less economic development had lower scores and, consequently, lower scores of adherence [12, 29, 30, 31, 34], which may be related to social and economic issues of the food system [25]. Sex, age and income are variables for which the most distinctions are found in different populations, as it is well established the adherence to this type of diet is greater among the female population, individuals with a higher income *per capita*, older age groups and residents of urban areas [29–31].

The lower adherence may be related to the contemporary eating pattern, with inadequate quantities of fruits, vegetables and plant-based proteins [35]. Similar findings were reported in studies conducted in Mexico and India [36, 37]. The stimulation of plant-based foods, which involve a lower generation of GHGE, could result in an increase in cost for some population groups [38]. Indeed, there is evidence that the EAT-Lancet diet is beyond the reach of needy families because nutrient-rich foods tend to come with a higher price tag compared to starchy staples and items rich in sugar and fat [39], which explains the lower adherence in developing countries [40]. Kesse-Guyot et al. (2021) pointed out the affordability of a sustainable and healthy diet, they found that participants with high EAT-Lancet adherence had the highest diet cost [25]. However the EAT-Lancet report it largely deals with the need to make a healthy and sustainable diet accessible to everyone from an economic point of view and gives suggestions to how to make it real [5]. There is also the issue of the stigmatization of this plant-based eating pattern for disrupting social conventions related to food, as the fear of stigmatization can be a barrier to avoiding meat consumption, which can be an obstacle to implementing the Eat-lancet diet [41, 42]. A healthy, environmentally sustainable diet should be accessible, which could limit indices that do not consider the cost of food [12].

Regarding structure among the indices analyzed, the ELD-1 and NB-EAT use a binary score, with 1 attributed if the participant met a goal and 0 if the goal was not met; the result is the sum of the nutritional goals met [18, 20, 25, 27, 29, 32]. The SHED index records agreement on a Likert scale ranging from 1 to 4 [11], whereas the EAT, WISH, PHDI and SDI have a gradual score proportional to the consumption of foods [3, 12, 19, 26, 28, 30, 31]. This method is encouraged by the EAT-Lancet report for the obtainment of intermediate values, which are common and more faithful for the assessment of food intake [5]. Food intake was addressed mainly in groups. Only the NB-EAT was based on nutrients [20]. The use of interchangeable groups is encouraged in the creation of indices based on the EAT-Lancet Commission. However, the use of nutrients enables a less precise assessment of adherence to these recommendations due to the provision of nutrient composition values of sustainable diets in a more restrictive, less informative way [5].

To define environmental sustainability indicators in the food sector, it is necessary to consider the different environmental dimensions impacted, such as GHGE, water resource use, land degradation and biodiversity loss [7, 9]. Among the main outcomes analyzed, the anthropogenic emission of GHGE was the most reported component and several researchers found a positive correlation between the total GHGE related to the diet and total energy and grams of the foods consumed [3, 12, 18, 19, 25–32]. Only the SHED index [11] is limited in this respect, as it does not quantify GHGs, which is the main metric to assess environmental burden, but by building on the EAT-Lancet reports, the SHED index assesses environmental burden indirectly by encouraging the consumption of a more plant-based and red meat-restricted diet to achieve the United Nations 2030 sustainable development goals and alleviate the burden of non-communicable diseases [5, 33, 43, 44].

Food production affects the environment and vice versa, as high environmental GHGE loads and unsustainable use of natural capital, such as overexploitation of natural resources and degradation of ecosystems, leads to environmental problems that have direct repercussions on climate instability. This climate instability, in turn, affects food production, creating additional challenges for food and nutrition security for future generations [6, 41].

The benefits of sustainable diets to human health are well established [41]. The use of different indices for the assessment of sustainable diets obtained positive outcomes for ischemic heart disease and diabetes [18, 27], the risk of weight gain, overweight and obesity [19, 31] and a significant reduction in the risk of cancer and cardiovascular disease [18, 34]. Knuppel et al. [18] found no clear association with mortality, whereas Stubbendorff et al. [28] demonstrated a 25% lower risk of mortality among individuals with greater adherence to the EAT-Lancet diet.

The offer of animal protein conditioned to essential human needs could reduce global morbidity and mortality rates associated with chronic diseases [20]. This would diminish the environmental impact of food production and would be capable of maintaining sustainability linked to the consumption of fresh, seasonal products produced locally and with minimal packaging [41]. It is possible that a balanced diet rich in vegetables is ideal, as the Western eating pattern and contemporary food systems place the health of both humans and the planet at risk [45, 46].

The indices analyzed have limitations that should be considered, such as gaps in the measurement of social dimensions and an intense focus on health (62% of sample), quality of diet (86% of sample) and environmental aspects (93% of sample), this suggests that there is an imbalance in studies regarding the different aspects of sustainability that are rarely recognized or comprehensively assessed when it comes to diets. Social aspects are critical to understanding the capacity of societies to engage in the definition and assessment of who wins and who loses as diets and food systems advance in response to the concerns of sustainability [7]. Previous reviews reported similar findings [7, 41, 47], demonstrating a lack of consideration for such issues in studies.

Another limitation was the lack of uniformity of the indicators assessed in the measurement of sustainable diets. However, this could also be considered an advantage when summarized scores are combined and may be applicable for use in population-based epidemiological studies. The sample size and quality of the papers ensure representativeness and confer reliability to the results, suggesting adequate quality of the evidence presented.

The inclusion and association of other indicators are suggested for the assessment of sustainable diets in indices that were not addressed here, such as changes in food transportation habits that can contribute to rural development and local production, which would consequently would contribute to a reduction in GHGE and global warming [48]. Transportation activities currently account for 23% of CO_2_ emissions, which is expected to double by the year 2050 [49].

This review also has strong points, such as the originality of the study with regards to the investigation of updated methods with a planetary scope in sustainable diet indices based on the EAT-Lancet Commission Summary Report. To the best of our knowledge, this is a pioneering synthesis on a subject that is becoming increasingly important. Lastly, a rigorous selection of papers was performed using pre-established inclusion criteria and the results can assist in the planning of public policies and orientations regarding the benefits of a healthy, sustainable diet based on the proposal of the EAT-Lancet Commission.

The findings of the present scoping review underscore the importance of studying indices that summarize components of complex modeling, which, when combined, present integrated structures that can explain the inherent relevance to the concept of healthy, sustainable diets.

There is an increasing need to improve indices of healthy, sustainable diets in the search for well-defined measures for the faithful assessment of the adherence of populations to sustainable diets. The EAT-Lancet Commission proposes scientific goals for these changes that should be reached by 2050 and such knowledge could assist in the adoption of measures that have a positive impact on the transformation of the long-term scenario of sustainability.

## 5. Conclusions

The evidence from this review highlights the diverse approaches employed by researchers to define sustainable diets based on the recommendations of the EAT-Lancet report. Our analysis significantly contributes to the current body of knowledge, presenting a description of the indexes that evaluate sustainable diets and indicators frequently used in the analyzes of articles (environmental and health). We identified serious barriers that hinder progress towards healthy and sustainable diets, including the difficulty of comparing different indices, the tendency to neglect social aspects and the lack of common definitions and metrics. Despite being challenging, the description of an ’ideal’ index that harmonizes the various indicators, promoting positive changes towards a more sustainable future is highlighted.

## Supporting information

S1 File PRISMA-ScR checklist. (DOCX)

S2 File Quality assessment for cohort studies (Newcastle-Ottawa Quality Assessment Scale criteria)*. (DOCX)

S3 File Quality assessment for cross-sectional studies (Modified Newcastle-Ottawa Quality Assessment Scale criteria)*. (DOCX)
