# Supplementary material for: Indices for measurement of sustainable diets: A scoping review
Source: PLoS One. 2023 Dec 20;18(12):e0296026. doi: 10.1371/journal.pone.0296026 (PMC10732454; doi:10.1371/journal.pone.0296026)
Supplement: S2 File — (DOCX) [file pone.0296026.s002.docx]

**S2 File. Quality assessment for cohort studies (Newcastle-Ottawa Quality Assessment Scale criteria)*.**

| First Authors surname (Year) | Selection  (max 4 stars) | | | | Comparability  (max 2 stars) | Outcome  (max 3 stars) | | | Final Score | Classification^a^ |
| --- | --- | --- | --- | --- | --- | --- | --- | --- | --- | --- |
|  | Representative sample | Selection of the non-exposed cohort | Ascertainment of exposure | Demonstration that outcome of interest was not present at start of study | Comparability of cohorts on the basis of the design or analysis controlled for confounders | Assessment of outcome | Was follow-up long enough for outcomes to occur | Adequacy of follow-up of cohorts described |  |  |
| Knuppel et al, 2019 [18] | ★ | ★ | ★ | - | ★ | ★ | ★ | ★ | ★★★★★★★ | Good |
| Seconda et al, 2020 [19] | ★ | ★ | - | ★ | ★ | - | ★ | ★ | ★★★★★★ | Good |
| Seconda et al, 2020 [26] | ★ | ★ | - | ★ | ★ | - | ★ | ★ | ★★★★★★ | Good |
| Kesse-Guyot et al, 2021 [25] | ★ | ★ | - | ★ | ★ | - | ★ | ★ | ★★★★★★ | Good |
| Xu et al, 2022 [27] | ★ | ★ | ★ | ★ | ★ | ★ | ★ | ★ | ★★★★★★★★ | Good |
| Stubbendorff et al, 2022 [28] | ★ | ★ | ★ | ★ | ★ | ★ | ★ | ★ | ★★★★★★★★ | Good |

^a^Quality score: Overall scores given (good, fair, and poor). Good quality: 3 or 4 stars (★) in selection domain AND 1 or 2 stars in comparability domain AND 2 or 3 stars in outcome domain; Fair quality: 2 stars in selection domain AND 1 or 2 stars in comparability domain AND 2 or 3 stars in outcome/exposure domain; poor quality: 0 or 1 star in selection domain OR 0 stars in comparability domain OR 0 or 1 star in outcome/exposure domain.

*Wells et al.,2014 [22].
