# Supplementary material for: Indices for measurement of sustainable diets: A scoping review
Source: PLoS One. 2023 Dec 20;18(12):e0296026. doi: 10.1371/journal.pone.0296026 (PMC10732454; doi:10.1371/journal.pone.0296026)
Supplement: S3 File — (DOCX) [file pone.0296026.s003.docx]

**S3 File. Quality assessment for cross-sectional studies (Modified Newcastle-Ottawa Quality Assessment Scale criteria)*.**

| First Authors surname (Year) | Selection  (max 4 stars) | | | | Comparability  (max 2 stars) | | Outcome  (max 2 stars) | | Final Score | Classification^a^ |
| --- | --- | --- | --- | --- | --- | --- | --- | --- | --- | --- |
|  | Representative sample | Sample size justified | Response rate satisfactory | Ascertainment of exposure (validated measurement tool used) | The study controls for disease severity | Study controls for other confounding factors | Assessment of outcome | Appropriate statistical test  described |  |  |
| Llanaj et al, 2021 [20] | - | ★ | - | ★ | - | ★ | ★ | ★ | ★★★★★ | Fair |
| Llanaj et al, 2021 [29] | - | ★ | - | ★ | - | ★ | ★ | ★ | ★★★★★ | Fair |
| Trijsburg et al, 2021 [12] | ★ | ★ | - | ★ | - | ★ | ★ | ★ | ★★★★★★ | Good |
| Cacau et al, 2021 [3] | ★ | ★ | - | ★ | - | ★ | ★ | ★ | ★★★★★★ | Good |
| Cacau et al, 2021 [31] | ★ | ★ | - | ★ | - | ★ | ★ | ★ | ★★★★★★ | Good |
| Hanley-Cook et al, 2021 [32] | ★ | ★ | - | ★ | - | ★ | ★ | ★ | ★★★★★★ | Good |
| Tepper et al, 2021 [11] | ★ | ★ | - | ★ | - | ★ | ★ | ★ | ★★★★★★ | Good |
| Marchioni et al, 2022 [30] | ★ | ★ | - | ★ | - | ★ | ★ | ★ | ★★★★★★ | Good |

^a^Quality score: Overall scores given (good, fair, and poor). Good quality: 3 or 4 stars (★) in selection domain AND 1 or 2 stars in comparability domain AND 1 or 2 stars in outcome domain; Fair quality: 2 stars in selection domain AND 1 or 2 stars in comparability domain AND 1 or 2 stars in outcome/exposure domain; poor quality: 0 or 1 star in selection OR 0 stars in comparability domain OR 0 or 1 star in outcome/exposure domain.

*Wells et al.,2012 [23].
